# Supporting mental health and psychosocial wellbeing through social and emotional learning: A participatory study of conflict-affected youth resettled to the U.S.

**DOI:** 10.1186/s12889-021-11674-z

**Published:** 2021-09-06

**Authors:** Lindsay Stark, Mackenzie V. Robinson, Alli Gillespie, Jeremy Aldrich, Wafa Hassan, Michael Wessells, Carine Allaf, Cyril Bennouna

**Affiliations:** 1 Brown School at Washington University in St. Louis, Campus Box 1196, One Brookings Drive, St. Louis, MO 63130 USA; 2 Harrisonburg City Public Schools, Harrisonburg, USA; 3 Global Educational Excellence, Ann Arbor, USA; 4 Mailman School of Public Health, Columbia University, New York, USA; 5 Qatar Foundation International, Washington, D.C., USA; 6 Department of Political Science, Brown University, Providence, USA

**Keywords:** Adolescents, Social emotional learning, Mental health, Psychosocial wellbeing, Refugees, Participatory ranking methodology

## Abstract

**Background:**

A growing literature has drawn attention to the central role that schools play in supporting the adjustment of resettled refugee youth and promoting their mental health and psychosocial wellbeing. In particular, the recent proliferation of school-based social and emotional learning (SEL) initiatives presents an opportunity to strengthen supports for resettled adolescents. This participatory research study aims to understand how high school students resettled from countries in the Middle East and North Africa region are experiencing the challenges and opportunities of acculturation and the ways in which they believe schools can better support them in this process.

**Methods:**

We analyzed primary data collected during focus group discussions as part of the SALaMA study. During these discussions, we used participatory ranking methodology to elicit adolescents’ suggestions on how high schools can better support students both academically and psychosocially after resettlement. Fourteen focus group discussions were held with male (*n* = 38) and female (*n* = 31) adolescents aged 14–20 years, who were selected purposively across six public high schools in Harrisonburg, Virginia, Austin, Texas, and Detroit, Michigan. Participants offered suggestions and then ranked them in order of importance using consensus ranking.

**Results:**

Thematic analysis of the PRM results across sites produced a wealth of suggestions centered around three broad themes, namely: skills related to navigating social and academic challenges, culturally responsive teaching, and socially and culturally equitable learning environments.

**Conclusions:**

Findings reported illustrate limitations of the conventional, universal SEL model and shed light on how schools can adapt transformative SEL strategies to serve their students better, especially newcomers from conflict-affected countries.

## Background

The number of forcibly displaced persons worldwide was estimated to have reached nearly 80 million as of 2019, with 40% of these persons under the age of 18 [1]. The same year, over a quarter of the 24,800 refugees resettled in the U.S. had originally fled countries such as Iraq and Syria [2] in the Middle East and North Africa (MENA) region. Various studies have documented the heightened mental health and psychosocial wellbeing risks among forcibly displaced children and adolescents [3–5]. If left unattended, these risks may impact adolescents’ life course by compromising the development of problem-solving, coping, and relationship-building skills and disrupting identity formation [3, 4, 6–8]. Daily stressors and supports in the receiving country may further affect the mental health of adolescents after resettlement.

A growing literature has documented the central role that schools play in supporting the adjustment of resettled newcomers [9]. Part of this adjustment involves acculturation, or the “changes that take place as a result of contact with culturally dissimilar people, groups, and social influences” [10]. Acculturation is a bidirectional process through which newcomers and the receiving community make adjustments to one another [11], and schools can promote this process by bolstering positive coping mechanisms and facilitating increased access to social support, which have been shown to improve adolescent wellbeing [12]. In particular, schools can serve as critical points of care and referral for specialized mental health and psychosocial supports (MHPSS), helping to overcome numerous barriers to services, including stigmatized attitudes towards mental health, inadequate resources, and limited access to providers among resettled families [13–17].

The proliferation of school-based social and emotional learning (SEL) programs and policies in recent years, in the U.S. as well as a number of other refugee-receiving contexts [18, 19], presents an opportunity to strengthen supports for resettled newcomers. SEL may refer to any number of frameworks and interventions that utilize approaches within the non-cognitive domain, or skills outside the academic realm [20]. One of the most commonly used SEL frameworks in the U.S., the Collaborative for Academic, Social, and Emotional Learning’s (CASEL) “universal SEL” model, aims to enhance students’ core competencies in five areas: self-awareness, self-management, social awareness, relationship skills, and responsible decision-making [21, 22]. The specialized curriculum is designed to foster development in multiple domains of resilience, interpersonal conflict and anger management, relationship-building, and problem-solving. In addition to classroom components, the universal SEL model includes interventions focused on adult SEL, school climate, and partnerships with families and communities [23].

The evidence suggests that universal SEL promotes sustained positive mental health and psychosocial wellbeing among adolescents, including increased prosocial behaviors, reduced emotional distress, and improved academic performance [18, 24–26]. SEL programming may provide a unique opportunity to create a more supportive environment for newcomers, and especially those arriving from conflict-affected contexts, who may benefit from their school’s attention to values such as building social awareness and developing positive, caring relationships among students and school staff [27, 28].

Despite its potential for supporting acculturation, the conventional SEL model has been criticized for its limited attention to the experiences, needs, and capacities of historically marginalized and racialized groups, including immigrants [29–31]. Critics have argued that SEL tends to focus too much on promoting student compliance with the dominant norms of acceptable emotional expression and social behavior, at times resembling a form of policing [32]. Critics also argue that conventional SEL neglects to engage students, and especially educators, in understanding and addressing critical dimensions of social and emotional equity, such as positionality, identity development, interpersonal bias, and racism [31–34]. Efforts to improve school climate, such as lowering academic expectations for students who are learning English or focusing solely on expanding students’ access to equal physical environments, have been seen as not doing enough to emphasize sociocultural equity and inclusion [35, 36].

In recent years, several efforts aimed at addressing the limitations of the conventional model have included a “transformative SEL” approach, which incorporates the development of positive ethnic-racial identity [37] and conducts critical analyses of privilege and power within the five SEL competencies [31, 38–41]. Transformative SEL also tends to be more aligned with MHPSS frameworks developed for use with conflict-affected populations [42, 43]. These frameworks place importance on building young people’s sense of identity to enhance self-efficacy and belonging. They also recognize youth voice and participation and the value of cultural resources in fostering wellbeing holistically [43].

The inclusion of these transformative SEL inputs substantially improves on the conventional SEL model, increasing its potential relevance to the experiences of student newcomers, like the ones in this study, from conflict-affected countries such as Iraq, Syria, and Yemen. Greater attention to the lived experiences, perspectives, and ideas of these young people may provide critical insight for making school-based supports more inclusive of refugee adolescents. We used participatory research methodologies in this study to understand how newcomer students from conflict-affected countries in the MENA region experience the challenges and opportunities of acculturation. Guided by the emerging transformative SEL framework, we also elicited students’ interpretations, ideas, and priorities regarding how their schools can better support them in this adjustment process. This paper adds to the current literature by centering student perspectives on how schools can more effectively serve the social, emotional, and academic needs of newcomer adolescents from the MENA region.

## Methods

### Setting

Data were collected as part of the Study of Adolescent Lives after Migration to America (SALaMA) to assess the mental health and psychosocial wellbeing of adolescents from the MENA region resettled in the U.S. [44–48]. The study was conducted in six public high schools across three sites: Harrisonburg, Virginia; Austin, Texas; and the Detroit Metropolitan Area (DMA), Michigan. These sites were selected purposively based on pre-existing collaborations, a history of resettling refugees from the MENA region, and interest from local school systems [44].

Collectively, Virginia, Texas, and Michigan resettled 123,288 refugees in the U.S. between 2008 and mid-year 2020 [49]. In 2015, 57.8% of refugees and special immigrant visa (SIV) holders^1^ in Michigan were from the MENA region, compared to 29.5% in Virginia, 20.2% in Texas, and 15.6% nationally [50].

Today, Harrisonburg City Public Schools (HCPS) serves around 6400 students, 46% of whom were born outside the U.S. Around 9% of HCPS’s student population speaks Arabic and 6% speak Kurdish [51]. MENA newcomers to Harrisonburg have most commonly included Iraqis and Kurds, whether as refugees or special immigration visa holders, as well as Sudanese and Syrian refugees. Harrisonburg began resettling refugees in 1988 and has served as a hub for immigrant populations ever since, with its generally welcoming policies towards newcomers earning it the moniker “The Friendly City” [52, 53]. In keeping with patterns in many parts of the U.S., adult refugees resettled in Harrisonburg are often employed in poultry processing, hospitality, or retail [54]. The local refugee resettlement office, Church World Service, helps resettled families to secure these jobs, as well as housing and access to social services. As for resettled youth, HCPS has developed and implemented a robust newcomer program that includes an orientation to life and school in the U.S., concentrated lessons on English and “newcomer survival skills,” and sheltered elective classes.

Due to a high cost of living and urban sprawl in Austin as compared to other cities, resettled refugees often live in low income or government subsidized housing and depend on federal resettlement and financial assistance [55]. Families from Syria, Iraq, and Sudan are among the MENA refugees who have resettled in Austin [55, 56], and adults often work in restaurants and small shops, as well as in their own home-based catering and sewing businesses [55]. While the state of Texas cut federal support for refugees in 2016, the city of Austin remains committed to resettlement [55]. Austin Independent School District (AISD) serves around 80,000 students, 27% of whom are English language learners (ELLs) with Arabic being the most common language after Spanish [57]. Schools offer targeted English support for newcomer students, including providing special “paraprofessionals” dedicated to assisting newcomer students in their classes with English translation and one-on-one tutoring.

While Harrisonburg and Austin are leaders in their states for refugee resettlement, the DMA was selected as a key site because it is well-known as having among the largest Arab ethnic enclaves in the U.S. [58]. Beginning with the arrival of Lebanese and Syrian merchants at the end of the nineteenth century, several waves of immigrants from the MENA region were drawn to the Detroit area in subsequent decades, largely due to its booming automotive industry [59]. Today, the Arab Community Center for Economic and Social Services (ACCESS) aids in the resettlement of forcibly displaced populations from conflicted-affected countries such as Iraq, Syria, and Yemen, as well as for refugees who chose to relocate to the DMA’s Arab enclave after originally settling in other U.S. cities [59]. Adolescent participants attended one of three Global Educational Excellence (GEE) charter high schools in the DMA, wherein about 53% of students are English Language Learners, with a significant percentage of students being resettled refugees or asylum-seekers from conflict-affected countries in the MENA region [60, 61]. In fact, students in GEE schools are required to take Arabic language courses, which aim to “enhance cultural awareness,” provide an opportunity to reinforce many students’ first language, and prepare students for life in a global society [62].

### Participants

Adolescent students (aged 14 to 20 years) were selected purposively at each school to participate in gender-stratified focus group discussions (FGDs) (Table 1). School-based staff assisted the research team in identifying and recruiting students who had arrived from conflicted-affected MENA countries in the past 5 years. Due to the unique demographics of the population in Michigan, we included some adolescent participants who were born in the U.S. or who had been in the U.S. for longer than 5 years, but whose families had emigrated from the MENA region in order to better understand wellbeing differences across time and between first and second immigrant generations. Further information on participant selection and recruitment is available elsewhere [44]. All FGDs in Austin and Harrisonburg were held exclusively with students born outside of the U.S. Across sites, the number of participants in each session ranged from three to eight.

**Table 1:** Sample demographics

Location	Austin	Harrisonburg	Michigan	All Sites
	***n***	***n***	***n***	***n (%)***
**# FGDs**	**3**	**3**	**8**	**14 (100)**
**Sex** (Age)				
Female (14–20)	3	9	19	31 (44.9)
Male (14–19)	10	8	20	38 (55.1)
**Country of Origin**				
Iraq	9	12	3	24 (34.8)
Syria	3	3	9	15 (21.7)
Yemen	0	0	12	12 (17.4)
United States	0	0	10	10 (14.5)
Jordan	0	0	3	3 (4.3)
Sudan	1	2	0	3 (4.3)
Palestine	0	0	2	2 (2.9)
**Total Participants**	**13**	**17**	**39**	**69 (100)**

### Data collection

Informed consent was obtained from all participants. For participants under the age of 18, written parental informed consent was obtained prior to approaching students for written informed assent. Students 18 years or older consented directly. Two trained facilitators led each focus group discussion. Participants were informed that the activity would take approximately 1.5 h and that all identifying information would be omitted during transcription. All group discussions were audio-recorded and took place at the schools in private rooms made available by school staff.

While most students participated in English, an Arabic interpreter was present as needed. The researchers engaged with a certified, local Arabic interpreter in all research sites. All three interpreters were foreign-born women, trained in interpreting for qualitative research technique and ethical research practices, and all had previously worked with adolescents. In each site, the research team also trained the interpreter in the research protocol, with a particular focus on research ethics for conducting data collection with conflict-affected youth [63, 64]. The interpreters and researchers also reviewed data collection instruments together in depth and the interpreters helped to localize lines of questioning and word choice. During data collection, all three provided verbatim interpretation and also helped to contextualize participant responses, when necessary. After each session, the researchers and interpreters met to debrief and to clarify any meanings behind certain statements that may not have been understood fully during the session.

Fourteen group discussions were held with a total of 69 adolescent participants representing nine countries in the MENA region. Exercises commenced with a discussion of students’ experiences at school and home using a semi-structured discussion guide, including broad framing questions such as, *“How does a new student at this school make friends?”* and *“How would you describe your relationships with teachers and other school staff?”*

In Harrisonburg and Austin, one facilitator was male while the other was female. In Michigan, the FGDs were facilitated by two females. For all FGDs, one facilitator took notes while the other facilitated the discussion around topics set forth in the FGD guide. Following this discussion, facilitators initiated a participatory ranking method (PRM) exercise [65]. Evidence highlights PRM as an effective consensus methodology with children and adolescents; the approach elicits opinions and ideas to ensure that intended outcomes are in line with participant priorities [66]. Participants were invited to answer the question “*How can high schools better support students who have been resettled from conflict-affected, Arab-majority countries?*” by free-listing suggestions for improved academic and psychosocial supports. The facilitators encouraged open discussion and confirmed there was sufficient agreement among group members. Once confirmed, a participant volunteer wrote a short, representative phrase agreed upon by the group for each suggestion. Facilitators validated participants’ interpretation of each concept before proceeding to the subsequent suggestion. Once the group had decided on a maximum of seven suggestions, they were asked to rank them collectively from ‘very important’ to ‘least important.’ Participants were asked to explain the decisions behind their comparative rankings and adjust until consensus was reached. The note-taker recorded participant dialogue throughout the processes of free-listing and ranking.

### Analysis

Using a grounded theory approach [67], the researchers used a combination of inductive and deductive analysis. The team alternated continuously between the empirical data and the literature to generate inferences that were at once theoretically informed and grounded in the language and expressed meanings of the participants. Two coders reviewed the PRM rankings and associated transcripts to conduct this thematic content analysis.

Recognizing that participant contributions extended well beyond the core competencies of the conventional SEL models, the researchers continued to review the social and emotional learning literature to identify frameworks and concepts that related more closely to student perspectives and priorities. Seven common themes emerged from this iterative analysis, and the researchers labelled these themes using the most relevant language from the literature. CASEL’s concept of schoolwide SEL then allowed us to organize these results around three school levels, including student competencies, adult SEL, and school climate. Once the common themes were established, coders categorized results according to these themes, with frequency and mean rankings calculated, with “one” indicating the highest priority. The researchers also drew on transcripts from the FGDs to inform the thematic content analysis, elucidate meaning, and compare prioritized supports across study sites.

### Ethics

Multiple measures were taken to ensure that the research was conducted in an ethical manner, with particular attention to securing voluntary, safe, and meaningful participation [45, 68]. Participants were offered a $20 gift card for their time and input upon completion of the FGD.

The study was reviewed and approved through the Columbia University Medical Center IRB (IRB-AAAR7830), the Washington University in St. Louis IRB (IRB-201905151), AISD’s Department of Research and Evaluation (R18.62), the Superintendent of Schools at HCPS, and the Director of GEE Schools. All methods were carried out in accordance with relevant guidelines and regulations.

## Results

Analysis of the PRM results across sites produced a wealth of suggestions pertaining to seven main themes of support, which correspond closely to the CASEL model and underscore the relevance of several elements of transformative SEL to the lives of newcomer students (see Table 2). These themes tended to cluster within three core elements of the SEL framework, namely: 1) Student SEL Competencies, 2) Adult SEL, and 3) School Climate.

**Table 2:** School support themes elicited during PRM exercises

SEL Level	Theme	Frequency	Mean Rank
**Student SEL Competencies**	**English Language Support** Ways to enhance English comprehension and communication skills as a pathway to integration and advancement.	14	2.8
	**Peer Support** Newcomer students desire to develop positive relationships with peers (both other newcomer and U.S.-born students).	10	2.9
**Adult SEL**	**Psychosocial Wellbeing** Ways in which schools as a whole promote resiliency among newcomer students.	8	2.6
	**Teacher Support** Key attributes of teachers and staff to instill confidence, trust, and foster success among newcomer students.	8	3.0
**School Climate**	**Orientation to Rules and Norms** Communicating vital information, including information on social norms, school rules, systems and operations, and available resources.	12	3.1
	**Sense of School Belonging** Ways for schools to enhance newcomers’ sense of belonging.	6	3.2
	**Cultural Responsiveness** The quality of school support related to social identity, language, customs, beliefs, and values identified by newcomer students as helping them to feel welcome, to acclimate, and to succeed.	6	3.2

### Student SEL competencies

Participants identified two primary pathways through which schools supported young peoples’ identity formation, self-efficacy, and sense of belonging—central principles of transformative SEL. First, students emphasized the process of developing a positive self-image through language acquisition. Second, students felt a sense of belonging that resulted from strengthened peer support networks.

#### Confidence and positive self-image through English language acquisition

Newcomer participants described how enhancing their ability to identify their strengths and develop social confidence and self-esteem allowed them to progress both academically and socially. This strengthened view of self was often tied to the success of English language acquisition. One student explained that building the “*confidence to learn English*” allowed him and his peers to *“introduce themselves*” and “*do activities where they could interact with other people*” (Michigan, Boy). Another student articulated that switching from primarily English as a Second Language (ESL) classes into regular classes encouraged him to “*speak out now*” instead of “*jus*t [hiding] *in your shadows*” (Harrisonburg, Boy). At the same time, a negative self-conception was sometimes tied to the ongoing challenges of language acquisition. One student explained this association:

So, let’s say that the teacher picks on the person that doesn’t really know how to speak English and he pronounces the word wrong, they’ll just start laughing. Yeah, it’s just sticky. And the dude doesn’t raise his hand up for the rest of the hour. Kinda ruins their self-esteem. (Michigan, Boy)

Students emphasized how language acquisition promoted an ability to regulate self-defeating emotions and manage stress associated with acculturation, helping them to develop and achieve goals. One student described how she pushed herself to accelerate English language learning as her teacher taught her more vocabulary. While at first “*people* [were] *rude to me since I was the only Arabic girl*,” she said, language acquisition empowered her to “*speak up against them,*” making her feel “*a lot better now*” (Austin, Girl). Another student mentioned that he and other students “*learn English through robotic*s,” which “*gave us something very big to dream, bigger than we can”* (Michigan, Boy). English language support resonated strongly with students as a key skill to build confidence and to promote growth and was notably the only theme identified and discussed in every focus group conducted.

#### Supportive peer relationships

The desire to develop positive relationships with peers was the third-most frequent theme arising from the participatory ranking activity. Newcomer students described feeling supported when schools fostered relationships with other newcomers from the MENA region. For example, a student who had been in the U.S. for several years described being asked by a counselor to speak with a recently arrived middle schooler who “*was lost*” and “*needed someone to explain everything.*” A week after the older student reassured the newcomer that “*it’s gonna be fine, I was like you,*” the counselor called to let her know that the newcomer was “*better … and talking to people more*” (Austin, Girl). Foreign-born students who had been in the U.S. longer often took it upon themselves to help more recent arrivals feel welcome, make new friends, and identify academic and non-academic supports. A student in Michigan’s Arab enclave, for instance, described how he served as a bridge for recently arrived newcomer students:

Even for the kids that, uh, came from overseas after us, and they don’t know how to speak English, we used to take them with us and chill with them and introduce them to other kids who don’t even speak English so they would feel comfortable. First day, second day, third day of school … they would be like friends with like, half of the school. (Michigan, Boy)

Students attested to the importance of being able to communicate effectively and meaningfully with peers, either in their native tongue or in English. As one newcomer student from Yemen attested, “*I imagine when I have language, I go right to* [other students] *and talk to them if I want anything, but I’m shy a little bit because I don’t have the language* (Michigan, Girl). Many newcomer students appreciated when U.S.-born students were curious about their culture in a way that was friendly and respectful, especially in Harrisonburg and Austin where MENA identities were less represented in the student body. For example, one participant mentioned “*a lot of people telling me ‘I like your hijab, like the way you wear it’,*” with some asking “*how did you come to here and where are you from …* [to] *start a conversation with me*” (Harrisonburg, Girl).

### Adult SEL and cultural responsiveness

Students emphasized the importance of the social and emotional competence of their adult educators, and specifically their ability to recognize and be compassionate towards the particular challenges of acculturation. Not only did students identify the need for educators capable of delivering culturally responsive lessons and promoting inclusive learning environments, but they also highlighted educators as general psychosocial support resources for students. The two sub-sections below explore these themes further.

#### Supporting student mental health and psychosocial wellbeing

Students viewed educators as being influential in promoting students’ sense of psychosocial wellbeing. Indeed, psychosocial wellbeing was the highest ranked theme among the PRM results. Newcomers considered educators to be frontline psychosocial supports that could provide advice and guidance on how to cope with school stressors and challenges. One student described how providing U.S.-born students with “*cultural information*” about newcomers’ home countries, beyond just teaching about their histories of political conflict with the U.S., might increase knowledge and perceptions that other students “*care about you,*” but emphasized that “*it’s not gonna help you as much as the teacher’s support*” (Austin, Boy).

Self-awareness and empathy among educators played a central role in many students’ decisions to seek support. In focus groups, students often mentioned specific teachers whom they collectively agreed were dependable. In one such group, for example, a student explained how “*when you have a problem and you go to Miss _, she’s really into it and she’s trying to help us with it,*” elaborating that “*when you see that you can trust her, she could really help you*” (Harrisonburg, Girl).

In addition to identifying specific school personnel as sources of support, students also discussed the importance of particular faculty and staff positions. For example, students recognized the vital role of school counselors in developing positive coping mechanisms for the challenges they faced. Voicing a common sentiment, one student reported she would “*go to* [the counselor] *sometimes … when I have problems*” (Michigan, Girl). Another participant described counselors as “*the best thing ever*” and mentioned that her counselor would “*keep everything you say to herself, help you out, and make you feel better*” when she sought assistance with school and housing-related issues (Harrisonburg, Girl).

Even though many students saw the potential for multidimensional support from academic counselors, a number of students felt underserved by the few counselors accessible within their schools. One student in Harrisonburg felt that her “*counselor is never there*” and was “*always busy,*” despite the student “[working] *so hard to find her*” (Harrisonburg, Girl). Students in Austin shared this sentiment, explaining that “*counselors* [are] *so busy always,*” often directing students to “*go look at* [resources] *online*” because they “*don’t have time*” (Austin, Girl). Multiple participants mentioned a lack of counselors utilizing language interpretation services during their meetings, while one student found his counselor to be discouraging when he wanted to take an honors class, asking “*Are you sure you’re ready? You might fail the class.”* The student felt that his counselor had expected less of him, “*just because I didn’t speak English*” (Harrisonburg, Boy).

Participants voiced a desire for more proactive efforts from school staff to prevent bullying, which they considered to be one of the core threats to their wellbeing. One student suggested that teachers form “*some kind of protection group … that will talk to the person that you’re getting picked on by*” and “*show them what’s actually going on in that person’s life and how it’s affecting people, like how he’s acting before and how he’s acting now since he’s getting picked on”* (Austin, Girl). The student also emphasized the importance of promoting empathy, proposing that teachers ask perpetrators of bullying to consider, “*if they went to a foreign country and they had to go through that,* [would] *you want* [bullying] *to happen to them?*”

#### Culturally responsive learning environments

Students expressed a desire for teachers and school staff to maintain a responsive and respectful environment where they demonstrate their equity literacy, especially related to the acculturative challenges affecting MENA students. A student in Michigan — who had educators both with and without his same cultural heritage — appreciated when “*teachers get to know each other, sit down, talk to each other … they all merge into one relationship and that makes them all treat students the same way, in a respectful way*” (Michigan, Boy).

One student described a tense situation in one of her classes where the teacher attempted to quell an argument between her Iraqi and Kurdish classmates over the autonomous Kurdistan region in Iraq, a highly disputed territory that has been the subject of an independence struggle among Kurds:

I’ve seen a fight about culture. Between an Iraqi girl and a Kurdish girl in my class. When my teacher was talking about it, it led to a fight … They were saying, ‘Oh, I have [a] country and it’s like, it’s not [just] a name, but it’s there.’ So, they fought over that … The teacher, he was trying to explain to them first, that it’s true that there’s no country, but there’s still Kurdish people that exist that have language. It’s just that they don’t have a territory. And he told [them], ‘There’s no reason why you guys should fight’ … he talked to them after [class]. (Harrisonburg, Girl)

In this situation, the teacher attempted to intervene and to ensure both students felt heard and respected. Students expressed feeling discouraged and alienated when teachers employed culturally insensitive or assimilationist methods, such as enforcing an English-only classroom, shaming students for their English language level or accent, or drawing unwanted attention to a student’s perceived social identity. One student described how some teachers made it difficult for her and other newcomer students to participate in class by continually telling them “*‘Speak English! Speak English!’ but nobody can, so it’s really hard*” (Michigan, Girl). Another student mentioned how educators sometimes spoke condescendingly to newcomers, expressing that “*just because* [students] *don’t speak English, they feel like they have to act with them like kids*” rather than “*talking to us like an adult*” (Harrisonburg, Boy).

Some students appreciated tailored supports, including when teachers and staff offered accommodations for religious practices, such as “[cutting] *back on the homework*” and “[giving] *us easy assignments*” during the month of “*Ramadan, where Muslims fast for around 17 hours a day*” (Michigan, Boy). However, a few students reported feeling singled out as needing special protection by teachers and staff, who may have been trying to provide well-intentioned supports. For instance, students recalled how a classmate with a disability and a refugee background was treated at their school. One student said that “*the principal and the teachers would point him out, they would try to be nice, but they would give him special treatment that you could tell he didn’t want*” (Michigan, Girl), with another student responding that *“they know they’re special, but don’t make them feel* [ashamed or embarrassed] *in front of everybody*” (Michigan, Girl). These responses describe a fine line between accommodating newcomers in a welcoming manner, in which educators recognize and respond to newcomers’ preferences, and stigmatizing newcomers through what participants called “*special treatment*,” where educators impose their own understanding of what newcomers need.

### School climate and promoting school belonging

Students appreciated warm and welcoming environments in their new schools and emphasized structural factors that promoted such a climate. These factors aligned with two subthemes: positive reception from faculty and students and schoolwide programming measures. The following two subsections explore how students’ sense of school belonging varied based on these factors.

#### Welcoming school climate

Students valued when their schools cultivated a welcoming context of reception for newcomers and supported their adjustment to their new environment. Compared with students in Austin or Harrisonburg, students at schools in Michigan seemed to encounter a relatively more welcoming environment upon arrival to the U.S., likely due, at least in part, to the composition of the student body and the large number of Arabic-speaking teachers and staff. For example, a participant in Michigan described feeling connected with other students who “*know the feeling and will try to help you, they will try to pick you up,*” as well as staff who “*help us a lot*” and “*know how to speak Arabic*.” Such a welcoming climate made the school “*a great place to start, and I’m glad I started* [here]” (Michigan, Boy).

When asked how newcomer students made friends at their new school, many students across study sites described strong initial connections with other newcomers. One student stated that it was easier to make friends with other students “*from your same ethnicity,*” because “*you have many things in common, you feel more comfortable*” (Harrisonburg, Girl). That being said, students believed school environments that fostered friendships *across* the student body, and not just with students of similar backgrounds, to be an important determinant of the ease and rate of their acculturation.

When students felt there was clear division of friend groups based on background, they also revealed a diminished sense of belonging. One student expressed:

I’m saying after you learn English even, you’re gonna get bullied when you have that accent. You have that not a Black-looking person and not a white. You have that Asian look or Middle Eastern, especially Yemeni look. And it’s hard because you’re not accepted by the white people or the Black people. You’re not accepted by the Americans, because you don’t speak the language. You don’t get accepted by the Yemenis because you start changing now. You’re not a Yemeni anymore. (Michigan, Boy)

As expressed above, students did not always feel welcomed in their new schools. One participant described his own negative experience with his initial school environment, noting that “*nobody will actually stop me to meet*” and that “*everybody is staring at me, like oh my gosh*” (Austin, Boy). Another student noted that as a newcomer, “*you feel like you’re not human.*” He went on to explain that “*if you are not feeling welcome, then sometimes you’ll feel like it just hurt me this time, I wanna suicide*” (Austin, Boy), demonstrating the sometimes severe impact of school climate on individuals’ mental health.

#### Schoolwide measures

Formal initiatives also supported students’ sense of belonging within schools. For starters, providing critical information to newcomer students on social norms, school rules, operations, and available resources upon arrival was deemed vital for adjustment and for expanding academic and social opportunities. In fact, orientation to rules and norms was the second most frequent suggestion that students produced in participatory ranking activities. When communication of rules and norms was insufficient, a series of negative consequences followed: Students who had been placed in a lower grade during their initial enrollment after resettlement thought that their level of education was underappreciated; students were disciplined for behaviors they did not understand had violated the rules, such as speaking their primary language; and they missed opportunities to enroll in classes or extracurriculars that interested them and that could advance their educational and eventual career success. One student raised a series of questions that she felt she had to figure out on her own:

Why are we going to classes instead of teachers coming to us? Why do we have homeroom? How do we get most of the credits without having to go through 3 or 4 years of high school? How can we make those credits cut shorter so we can graduate earlier? Those questions, you kind of figure them out as you get older. Time fixes things. (Austin, Girl)

Another student mentioned being “*in a lot of trouble ‘cause I didn’t know* [all the new rules],” emphasizing that teaching newcomers these norms should be “*the first thing*” schools do (Michigan, Boy). Students appreciated initiatives such as Harrisonburg’s Peer Leader Program, in which students “*learn about each other and* [from] *where they came,*” (Harrisonburg, Boy), as these programs were thought to create a welcoming space for students to share experiences and information, exchange advice, and form relationships within the broader community.

Newcomer students also valued formal opportunities to access and share information about their countries of birth and cultural heritage in a manner that did not ostracize them, but rather recognized their dignity. These participants wanted schools and teachers to educate their classmates about the rich national histories of their countries of origin. One student expressed that learning “*about my country, about my culture*” made him feel like “*oh, they acknowledge me* … *they want to learn more about it so they can learn more about me.*” When teachers and peers made the effort to talk about his country out of “*all the countries in the world,*” it made him “*feel kinda special*” (Austin, Boy). A student in Michigan recalled a particular initiative his school took to promote cultural inclusion and appreciation, called “Multicultural Day:”

Each student picks a country … and you basically have to research the history, put it all on a poster and build something … I did Jordan. I built a soccer field, ‘cause their culture is soccer. Each year, you choose a different country and the day that we have to present, the whole school’s different. It’s filled with different cultures and each person that comes to your station, you present to them everything you learned and you show them the activity. (Michigan, Boy)

Rather than discussing the wars taking place in their home countries, students wanted more positive representation of their homelands reflected in curricula. When asked what such representation could look like, one student responded: “*I kind of don’t like the word ‘history,’ ‘cause it involves a lot of stuff, including* [a lot of] *wars. It’s not that fun. But giving* [other] *information can be interesting cause you’re talking about different cultures, not giving* [just] *history*” (Austin, Boy).

In addition to positive representation in curricula, students appreciated inclusion in classes and extracurricular activities. Although classroom programming for newcomers varied across study sites, a common practice involved placing recently arrived students in sheltered instruction for a given period to scaffold their language skills and content-area knowledge before integrating them into the general student body. In one PRM focus group activity, however, a student suggested that newcomers join the wider student body in regular, English-based classes instead of being “*separated*” from U.S.-born students throughout the day. The student explained that “*if you only put* [newcomer students] *all in one class, they won’t be able to* [experience] *their new life.*” She elaborated that full integration would “*help them gain confidence into opening up and into the new world*” (Michigan, Girl).

Further, newcomer students described feeling confident and accepted when they participated in student groups, teams, and clubs. As one student shared, *“everyone start* [ed] *picking me as their friend*” after his soccer coach selected him to play in a game (Austin, Boy). Another student highlighted how participation on the robotics team allowed him to achieve goals: “*we make a team together, we work together, and we have a reward last year for the* [fastest] *improving team in the last two years. We go from last to top 10”* (Michigan, Boy). Students were also members of cultural heritage groups. One student described joining his school’s “*Arab Student Association, so you can do sports in it and they can help you with student mentors*” (Michigan, Boy).

## Discussion

Over the course of their displacement and resettlement, conflict-affected adolescents may face countless stressful life events and acculturative stressors that heighten their risk of adverse mental health and psychosocial wellbeing outcomes. As the central institutional support in adolescent lives, schools play a crucial role in welcoming young newcomers and promoting their adjustment [3, 9]. Participants in this study felt that schools supported newcomers’ mental health and psychosocial wellbeing in numerous ways. They highlighted how schools fostered a positive self-image and promoted healthy peer relationships, how educators served as frontline psychosocial supports, and how administrations took a range of schoolwide measures to improve climate and students’ sense of belonging. Notwithstanding these considerable efforts, the participatory ranking approach empowered participants to share experiences and ideas that could extend and complement current supports. Enhanced English language support, orientation to rules and norms, peer and teacher support, and cultural responsiveness were all considered necessary for improving sense of school belonging and psychosocial wellbeing. While there was not a wide range in mean or median rankings, mental health and psychosocial supports emerged as the highest ranked theme and English support was the only theme mentioned across all groups.

The findings demonstrate the value of a transformative approach to SEL—with its emphasis on inclusion, equity, and positive ethnic-racial identity development [37]—in enhancing newcomer wellbeing. At the same time, the various perspectives of MENA youth also have the potential to help fill current gaps in the model in terms of its applicability to newcomer students. Beyond the competencies addressed in existing SEL models, students spoke to the crosscutting, deleterious effects of the challenges they encountered as newcomers that undermined their sense of self-efficacy and ability to manage acculturative stressors, including microaggressions [69] and discrimination. They emphasized the impact of culturally responsive teachers on their psychosocial wellbeing and appreciated when teachers connected them with other students and listened to their problems. Students also pointed to how teachers might modify their behavior to ensure positive student-educator relationships and inclusive learning environments. For example, assimilationist educational approaches that discouraged and even punished native language usage frequently made students in this study feel intimidated and demoralized. Evidence shows that educators who exhibit an ‘assimilationist’ stance in regard to newcomer students’ acculturation process, with the belief that they should strictly abide by the norms of their new school and the dominant culture of their new country, can have negative effects on sense of belonging and academic success [26, 58].

**Fig. 1:**
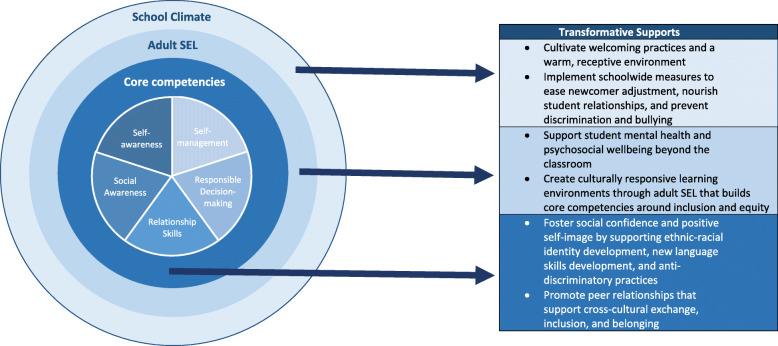
Transformative Supports for SEL Framework

Thus, our findings illuminate the need to expand the transformative SEL framework to devote more attention and resources to acculturation and cultural responsiveness at individual, classroom, and school levels. Figure 1 presents our adaptations to the SEL model to more fully represent the needs and expressed preferences of newcomer students. At the level of the individual, the framework should broaden to include language development skills and supports for positive ethnic-racial identity development and anti-discriminatory practice as means of fostering students’ social confidence and positive self-image. Further, transformative SEL could promote peer relationships that more explicitly nurture cross-cultural exchange, inclusion, and belonging. On the level of adult SEL, our findings suggest that teachers should extend mental health and psychosocial wellbeing support beyond their classroom and create culturally responsive learning environments within them. The findings also indicate the need for more concerted efforts to build adult SEL such that educators become more capable of assessing their own positionality and biases, and more capable of adjusting to the needs and preferences of students and their families. The cultivation of welcoming practices and schoolwide measures to ease newcomer students’ adjustment, including the prevention of discrimination and bullying, are vital components to augment the third SEL level, school climate.

On a broader scale, participants lauded schoolwide efforts to create and maintain systemic supports for newcomers and considered these measures a way to promote a greater sense of school belonging. Recognition of students’ cultural identities, without reducing them to their newcomer/refugee status, was deemed vital in mitigating feelings of ‘otherness’ within schools and fostering peer inclusion. Students responded positively when representations of their heritage cultures were presented as assets, rather than as flattened caricatures. Experts argue that inattention to matters of identity, belonging, and inequality in conventional schooling and in universal SEL has a variety of negative consequences, including increased acculturative stress and a compromised development of a positive ethnic-racial identity [31, 70]. Research suggests that a positive sense of ethnic-racial identity can help students to develop and employ “cultural repertoires” that allow them to build and sustain self-esteem and belonging, ultimately leading to resilience against marginalization [71]. Children and adolescents are at a greater advantage when developing these repertoires, as their position allows them to mold the social environment for future generations [65].

A comparison of findings across study sites particularly highlights the impact of positive ethnic-racial identity on wellbeing for newcomer adolescents resettled in Michigan’s ethnic enclave, where Arab cultural practices are embraced by schools and educators. Garner et al.’s reconfiguration of the SEL model emphasizes the role of a school’s sociocultural context, and placement within the community it serves, in promoting students’ SEL competencies [72]. Schools’ geographic location and racial and ethnic composition are considered “direct influencers” of student wellbeing, according to this model [72]. Whereas participants in Austin expressed difficulties when facing bullying from students from other racial/ethnic groups, analysis of peer support in Michigan reported elsewhere was found to include two-way benefits of friendships between U.S.-born and foreign-born adolescents with shared cultural heritage [46]. Findings also suggest that schools’ ability to provide safe environments, including bullying prevention, may be an important factor in creating a welcoming environment for newcomer students. Such factors and their influence on psychosocial wellbeing speak to the urgency of efforts to improve on the conventional universal SEL model and the importance of listening to the experiences and ideas of students from a wide range of backgrounds [33].

We recognize a few limitations with our study. Not all members of the research team are Arabic-speaking or have MENA heritage. While the team engaged in ongoing reflexive practice and conferred with the members of the research team with similar backgrounds to our participants, there may be some limitations in our own interpretation of the results. We also recognize the limitation in analyzing student perspectives alone, especially as social desirability may bias what adolescents choose to share in peer focus groups, though we are encouraged by how forthcoming students were about both negative and positive experiences in these schools. It is also valuable to consider the perspectives of educators, parents, and district officials to understand the full picture of current and future schoolwide measures aimed at supporting students’ mental health and psychosocial wellbeing. The findings presented in this paper are intended to be situated alongside our other analyses in order to provide broader insights into supporting the wellbeing of this population [28, 45–48]. Overall, student ideas and feedback reaffirm the critical importance of efforts to improve SEL’s attention to sociocultural equity, inclusion, and participation, principles that may be present to varying degrees across the wide range of SEL models and programs being used around the U.S. [20]. Students’ knowledge and feedback give insight as to how SEL improvements might help to ease processes of resettlement and acculturation. Further, the wide range of suggestions emerging from our participatory approach illuminates the value of expanding the evidence base on the direct preferences of adolescent refugee youth. These findings are by no means representative of the full range of perspectives and experiences among conflict-affected youth from the MENA region resettled in the U.S.

Future research should prioritize culturally responsive quantitative methods to evaluate the continuous impact of transformative SEL interventions on students’ identity development, agency, belonging, and, ultimately, mental health and psychosocial wellbeing [73, 74]. Further, evaluating key outcomes for teachers and school staff providing these curricula, such as empathy, cultural awareness, and self-awareness—all of which were identified by students as critical to their likelihood of seeking teacher support—can help to identify whether any additional professional development is needed to ensure SEL programs are implemented with fidelity. While fostering peer mentorships and friendships between newcomer and U.S.-born students with a shared cultural heritage (as was done in Michigan) may be difficult in schools with more culturally homogenous populations, school districts might consider implementing similar policies at the district level, with research examining whether benefits are still conferred when such relationships are cultivated across schools.

## Conclusion

While ample evidence supports the important role that schools play in promoting forcibly displaced adolescents’ mental health and psychosocial wellbeing, minimal literature to date has documented newcomer students’ perspectives and recommendations on how schools can serve them more effectively. The findings from this study emphasize the importance of including student voices in identifying ways to improve school supports for their ultimate benefit, especially for students from historically marginalized backgrounds. School initiatives based on the SEL models, and especially transformative SEL, show great promise in addressing gaps, though students illuminate the need for stronger inclusion of MHPSS, English language supports, and sociocultural equity with educators and peers. These findings may not only advance the refinement of the transformative SEL model, but also inform efforts to improve school-based psychosocial interventions for newcomer adolescents and ultimately students’ mental health and wellbeing.

## Data Availability

The datasets generated and/or analyzed during the current study are not publicly available due to IRB restrictions but are available from the corresponding author on reasonable request.

## Abbreviations

AISD: Austin Independent School District
CASEL: Collaborative for Academic, Social, and Emotional Learning
DMA: Detroit Metropolitan Area
FGD: Focus group discussion
GEE: Global Educational Excellence
HCPS: Harrisonburg City Public Schools
MENA: Middle East and North Africa
MHPSS: Mental health and psychosocial supports
PRM: Participatory ranking methodology
SALaMA: Study of Adolescent Lives after Migration to America
SEL: Social and emotional learning
SIV: Special Immigrant Visa
U.S.: United States

## Footnotes

1 Special immigrant visa holders are people who have supported or worked with the U.S. Armed Forces and as such, are eligible for resettlement benefits and programming upon arrival in the U.S.
